# Prevalence of Hypertension and Associated Factors among the Outpatient Department in Akaki Kality Subcity Health Centers, Addis Ababa, Ethiopia

**DOI:** 10.1155/2020/7960578

**Published:** 2020-08-18

**Authors:** Tadesse Bedada Haye, Berhanu Tolera Agama

**Affiliations:** Addis Ababa University College of Health Sciences, Addis Ababa, Ethiopia

## Abstract

**Background:**

Fatalities from hypertension in East Africa are increasing, even though they decreased in western industrial regions. Older age, being female, illiterate, smoking, physical inactivity, and high waist circumferences are major risk factors for the development of hypertension. The prevalence of hypertension among Federal Ministry Civil servants in Addis Ababa, Ethiopia, has found to be high; which is an indication for institution-based hypertension-screening programs.

**Objective:**

Prevalence of hypertension and associated factors among the outpatient department in Akaki Kality Subcity Health Centers, Addis Ababa, Ethiopia. *Methodology*. Facility-based cross-sectional study was carried out on systematically sampled 401 out-department patients whose age was greater than or equal to 18 years in four government health centers in Addis Ababa. Data collection took place from March 10, 2018, to April 06 2018. Binary logistic regression analysis was carried out to identify predictors of hypertension.

**Results:**

Patients had a mean age of 41.17 years (95% CI: 39.77–42.57). The prevalence of hypertension was 14% (95% CI: 13.653–14.347), and 30 (53.57%) were males. Alcohol drinkers were 11.844 times more likely to be hypertensive as compared to non-alcohol drinkers (AOR = 11.844, 95% CI: 3.596–39.014). Cigarette smokers were 16.511 times more likely to be hypertensive as compared to non-cigarette smokers (AOR = 16.511, 95% CI: 4.775–57.084). Khat chewers were 6.964 times more likely to be hypertensive as compared to non-khat chewers (AOR = 6.964, 95% CI: 1.773–26.889).

**Conclusion:**

The prevalence of patients with hypertension was 14%. Alcohol drinking, cigarette smoking, khat chewing, body mass index ≥25 kg/m^2^, and age ≥44 years old are major determinants identified by this study. Hence, appropriate management of patients focusing on the relevant associated factors would be of great benefit in controlling hypertension.

## 1. Background

Heart pumps blood around the body through blood vessels. Blood pressure is the force at which blood moves through these vessels [1]. Hypertension means systolic blood pressure greater than or equal to 140 mmhg and/or diastolic pressure greater than or equal to 90 mm hg [2]. Thirty-five million people died from noncommunicable diseases in the world, and 80% of the deaths were in lower- and middle-income countries [3]. According to a study that was conducted in the USA, among 33,086 study participants, maternal alcohol drinking during pregnancy was associated with the development of hypertension [4]. Noncommunicable diseases, including hypertension, are the leading causes of death among adults in Addis Ababa together with the existing burden of communicable diseases [1].

In the United Arab Emirates, the prevalence of hypertension in a representative sample of young male South Asian immigrants living in the UAE was relatively high. However, the awareness, treatment, and control of hypertension within this population were very low [5]. Trends in blood pressure show an increase in systolic blood pressure in many sub-Saharan regions, and these are among the highest in the world [6].

One study that was conducted in Malawi, among 952 participants, showed the prevalence of hypertension was 23.7% [7]. The prevalence of hypertension is found to be high; prevention strategies are urgently needed to address this life-threatening and important risk factor for cardiovascular diseases including hypertension [8].

The fatalities from hypertension in East Africa are increasing even though they decreased in the western industrial regions [9].

Since cardiovascular disease is by far the largest cause of years of life lost in developing countries, the need for new approaches towards control of this major risk factor should be emphasized [10]. In South Africa, it was revealed that the prevalence of hypertension was found to be 77.3% among older adults [11].

Noncommunicable diseases, including hypertension, are the leading causes of death among adults in Addis Ababa, together with the existing burden of communicable diseases [8].

Based on some studies, male sex, overweight, and sleeping <5 hours per day were the common factors for the development of hypertension [12–14]. In addition, male sex, having a family history of hypertension, inadequate physical activity, low consumption of vegetables, excess salt consumption, and obesity were among the factors [15].

Bankers and teachers in Addis Ababa had the prevalence of 21% hypertension, whereas community in Northwest Ethiopia had 27.9% [16, 17]. Urban communities and female gender are more affected by hypertension compared to rural communities and male gender, respectively [17]. The prevalence of hypertension among university students was 7.7% and recorded as the lowest prevalence of the studies [18].

Even though evidence shows high prevalence and different risk factors at different parts of the world as well as in Ethiopia, there are inadequate studies in Addis Ababa regarding hypertension and its determinants. Therefore, this study aimed to collect and organize the data about prevalence of hypertension and associated factors.

## 2. Methods

### 2.1. Study Area and Period

The study was conducted among outpatients in Akaki Kality Subcity four governmental health enters from March 10, 2018, to April 06 2018. There are seven government-owned and one nongovernmental health centers in the subcity.

Study design: institutional-based descriptive cross-sectional study design was used.

### 2.2. Populations

Target population: outpatient department attendants at the health center found in Akaki Kality Subcity.

Study population: outpatient department attendants from the four health centers in Akaki Kality Subcity.

### 2.3. Inclusion and Exclusion Criteria

Inclusion criteria: all adult outpatient department attendants.

Exclusion criteria: severe ill patients who were not able to respond.

### 2.4. Sampling Size Calculation and Sampling Procedure

The sample size was calculated using the formula for a single population proportion. Systematic sampling was used to select the size proportionate sample from each selected health center. Four health centers out of the seven were selected for the study. The total number of patients in four health centers during the study was 9246.

To calculate the sample size, according to the study that was conducted in Addis Ababa among the Federal Ministry of Civil servants in 2014, the proportion of hypertension was 27.3% and then *P*=0.0273, *d* = 0.05, confidence level = 95%, *n* = ((*Zα*/2)*pq*)/*d*^2^ = (1.96^2^ × 0.273 × 0.727)/0.0.05^2^ = 305.

The average risk factor of hypertension (smokers-53%, alcohol drinking-32.9%, and khat chewing-35.8%) was 40.57%.

*P*=0.4057, *q* = 1 − *p* = 1 − 0.4057 = 0.5943; then, *n* = (*Z*_*α*/2_)^2^_*Pq*/*d*_^2^ = (1.96)^2^ (0.4057 × 0.5943)/(0.05)^2^ = 370. Then, by adding 10% of nonresponse rate gives 407, which was the final sample size. To get the sampling interval, 9246/407 = 23; then, *K* is 23. Therefore, the study participants were selected every 23 intervals.

### 2.5. Study Variables

Dependent variable: prevalence of hypertension.

Independent variables: sociodemographic characteristics, alcohol consumption, khat chewing, cigarette smoking, and body mass index.

### 2.6. Data Collection Techniques

Face-to-face interview of the patients about sociodemographic characteristics, consisting of age, sex, marital status, income, and occupational status, was conducted by a trained interviewer. The questionnaires were adopted from the WHO STEPwise survey and included additional information. Moreover, blood pressure was measured using a manual sphygmomanometer and stethoscope, weight by using the adult weight scale, and height by using a tape meter. The questionnaires were prepared in English language and then translated into Amharic language and back-translated to English to check its consistency.

### 2.7. Data Processing and Analysis

The principal investigators cleaned and repeatedly checked data and entered the collected data to Epi Info version 7 for further organizing and processing. Then, data were transformed to SPSS version 20 in order to analyze them. Descriptive statistics, such as mean, median, and standard deviation, were calculated. Binary logistic regression was used to identify factors associated with hypertension. Lastly, the findings were organized, interpreted, and narrated accordingly.

### 2.8. Data Quality Control

The principal investigators checked the data during collection and analysis to ensure that all the information was properly collected and recorded. In addition, the information was checked again for completeness and internal consistency.

### 2.9. Operational Definitions

  Alcohol consumptions: drinking greater than 30 grams of ethanol per day
  Hypertension: systolic blood pressure ≥140 mmHg and or/diastolic blood pressure ≥90 mmHg
  Moderate drink of alcohol: two drinks a day for men <65 years old or one drink a day for age >65 years and women
  One drink of alcohol: 355 ml of beer or 148 ml of wine or 1.5 ml of sprite
  Physical activity: walking, jogging, swimming, and cycling at least 30 minutes per day

## 3. Results

Four hundred and one outpatient department attendants (with a response rate of 98.53%) participated in this study. The respondents were selected from four health centers found in Akaki Kality subcity of Addis Ababa City Administration.

A majority of the respondents were urban dwellers (381, 95%). Two hundred and thirty-one (57.6%) were females. Their mean age was 41.17 years (95% CI: 39.77–42.57). Three hundred and seven (76.6%) participants were Orthodox Christianity followers. One hundred and seventy-two (42.9%) respondents were Oromo ethnic group. Two hundred and seventy (67.3%) of them were married, and 113 (28.2%) were educated to the level of grade 1 to grade 8. One hundred and nine (27.2%) participants were government employees, and their mean monthly income was 2712.68 birr (95% CI: 2493.9–2931.46) (Table 1).

### 3.1. Anthropometric Values and Behavioral Characteristics of the Study Subjects

Mean value of weight, height, and body mass index of the participants was 67 kg (95% CI: 65.84–68.16), 1.65 meters (95% CI: 1.646–1.654), and 24.27 kg/m^2^ (95% CI: 23.93–24.61), respectively.

About forty-nine (12.2%) of participants smoke cigarettes, and twenty-five (6.2%) of them smoke one pack of cigarettes per day. One hundred and fifty-one (37.7%) and forty-five (11.2%) study participants drink alcohol and one drink weekly, respectively. Forty-four (11%) chew khat, and fifteen (3.7%) of them chewed khat daily. One hundred and ninety (47.7%) of study participants were practicing regular exercise, and one hundred and forty-six (36.4%) of study participants said walking was considered as a regular exercise (Table 2).

### 3.2. Status of Hypertension among Study Participants

The mean systolic blood pressure and diastolic blood pressure of study participants were 120.88 mmHg (95% CI: 119.26–122.50) and 79.87 mmHg (95% CI: 79.01–80.73), respectively. The overall prevalence of hypertension among study participant was 14% (95% CI: 13.653–14.347). Accordingly, ten (2.5%) outpatient department attendants have isolated systolic hypertension (≥140/<90 mmHg), eight (2%) have isolated diastolic hypertension (<140/≥90 mmHg), 33 (8.3%). Concerning the stage of hypertension, eighteen (4.5%) of study participants have stage I hypertension, and four (1%) of them have stage II hypertension (Table 3).

### 3.3. Factors Associated with Prevalence of Hypertension

In bivariate analysis, weight of study participants was significantly associated with hypertension (OR = 1.082; CI: 1.051–1.113) (*P* ≤ 0.001). However, height of study participants was not significantly associated with hypertension (*P*=0.829). Body mass index of study participants was significantly associated with hypertension (*P* ≤ 0.001). Alcohol drinkers were 32.303 times risky for hypertension than nondrinkers (OR = 32.303, 95% CI: 11.379–91.702, *P* ≤ 0.001). Cigarette smokers were 93.33 times risky for hypertension compared to nonsmokers (OR = 93.33, 95% CI: 38.713–225, *P* ≤ 0.001).

Khat chewers were 43.57 times risky for hypertension when compared to non-chat chewers (OR = 43.57, 95% CI: 19.52–97.21, *P* ≤ 0.001). Study participants practicing regular exercise were 1.137 times risky for hypertension as compared to those who practiced it regularly (OR = 1.137, 95% CI: 0.644–2, *P*=0.658). In the multivariate logistic regression analysis, drinking alcohol, cigarette smoking, khat chewing, body mass index, and age were significantly associated with the development of hypertension (*P* < 0.05).

Accordingly, alcohol drinkers were 11.844 times more likely to be hypertensive as compared to non-alcohol drinkers (AOR = 11.844, 95% CI: 3.596–39.014). Cigarette smokers were 16.511 times more likely to be hypertensive as compared to non-cigarette smokers (AOR = 16.511, 95% CI: 4.775–57.084). Khat chewers were 6.905 times more likely to be hypertensive as compared to non-khat chewers (AOR = 6.905, 95% CI: 1.773–26.889) (Table 4).

## 4. Discussion

Hypertension is becoming one of the major noncommunicable diseases in Ethiopia. It contributes significantly to high burden of heart diseases [17]. In this study, the overall prevalence of hypertension among outpatient attendants was 14.0%. It was almost similar with previous studies done in China (16.5%), Northwest Ethiopia (13.3%), and Addis Ababa (14.9%) [19–21]. However, this finding is higher than the study done in Gondar University, Ethiopia (7.7%). The reasons for low prevalence of hypertension among university students were low age distribution (mean 21 years), high prevalence of underweight (35.6%), and low body mass index (mean 19.8 kg/m^2^) [22].

The prevalence of hypertension among study participants is lower than studies done in Addis Ababa (27.3%), Kenya, Nairobi (29.4%), Bahir Dar (25.1%), Durame town in Ethiopia (22.4%), Northwest Ethiopia (27.9%), and many others. This could be because one of the previous studies used multistage sampling procedure to collect study participants [23]. The other possible differences were older age and large sample sizes in previous studies [24, 25].

Increased age was identified as a factor for hypertension in this study and other studies [5, 23, 26]. This could be due to physiological change of blood vessels as the age increases.

Unlike many other studies [20, 22, 27, 28], this finding did not reveal any association of hypertension with weight. This could be due to difference in the study population, setting, sample size, and socioeconomic and cultural differences between the study participants.

In this study, outpatient attendants who have been drinking alcohol were 11.844 times more likely to be hypertensive as compared to those who have not been drinking alcohol. It was similar with the studies conducted in Gondar in Ethiopia and other parts of the world [22, 23, 29]. The possible reasons are that alcohol can produce central nervous system imbalance (initiates both central and peripheral reactions which lead to hypertension), baro reflex impairment (baroreflex challenged vasoconstrictors such as phenyl-epiphrine and angiotensin II), sympathetic out flow (causes secretion of corticotrophin releasing hormones (mineralo-corticostroid and catecholamines)), rennin-angiotensin system initiation, expansion of extracellular fluid (elevation of plasma vasopressin and rennin activities), and then shifts extracellular calcium to intracellular space by increasing sensitivity to vasoconstrictor nor-epinephrine. These all lead to vasoconstriction and cause hypertension [12–15, 29].

History of smoking is one of the well-established risk factors for hypertension [12]. Similarly, in this study, smokers were 16.511 times more likely to be hypertensive as compared to nonsmokers. This study is similar with studies that were conducted in different parts of the world, including Ethiopia [14, 30–32]. Cigarette smoking causes activation of the sympathetic nervous system and oxidative stress associated with increase markers of inflammation leading to endothelial dysfunction, vascular injury, plague progression, and increased arterial stiffness leading to the development of hypertension [33–35]. Many researchers have reported that cigarette smoking is positively associated with hypertension [11, 21, 25, 36–38], which is similar to the result of our study that revealed cigarette smoking was significantly associated with hypertension. The possible reason might be that cigarette smoking increases arterial inflammation and stiffness [29].

According to this study, khat chewers were 6.964 times at risk for hypertension than non-khat chewers. Others study also confirmed positive association between khat chewing and development of hypertension [29, 39]. The possible reason for this association is that khat contains a chemical called cathinone, which is a potent vasoconstrictor that causes diastolic blood pressure elevation. This is the reason why khat chewers were more likely to be hypertensive when compared with non-khat chewers [40].

## 5. Conclusion

In conclusion, the proportion of patients with hypertension was considerably high (14%), which is higher than reports from many countries. The findings obtained from multivariate logistic regression analysis suggest that hypertension was associated with age ≥44 years old, body mass index ≥25 kg/m^2^, alcohol drinking, cigarette smoking, and khat chewing. Hence, appropriate management of patients focusing on the relevant associated factors identified for control of hypertension, which are modifiable factors, remains the mainstay to maintain control of hypertension.

## Figures and Tables

**Table 1:** Sociodemographic characteristics of the study subjects, Akaki Kality, Addis Ababa, Ethiopia, 2018.

Characteristic	Women, *n* (%)	Men, *n* (%)
Place of residence		
Rural	12 (5.2)	8 (4.7)
Urban	219 (94.8)	162 (95.3)
Age, year		
≤24	28 (12.1)	21(12.4)
25–34	65 (28.1)	37 (21.8)
35–44	61 (26.4)	42 (24.7)
45–54	43 (18.6)	27 (15.9)
55–64	21 (9.1)	24 (14.1)
≥65	13 (5.6)	19 (11.2)
Mean	41.17	
Religion		
Orthodox	178 (77.7)	129 (76.8)
Muslim	23 (10)	22 (13.1)
Protestant	27 (11.8)	13 (7.7)
Catholic	1 (0.4)	4 (2.4)
Others	2 (0.1)	0(0)
Ethnicity		
Oromo	100 (43.3)	72 (44.4)
Amhara	73 (31.6)	59 (34.7)
Tigre	33 (14.3)	19 (11.2)
Guraghe	21 (9.1)	14 (8.2)
Others	4 (1.7)	6 (1.5)
Marital status		
Single	22 (9.5)	26 (15.3)
Married	144 (62.3)	126 (74.1)
Widowed	34 (14.7)	6 (3.5)
Divorced	25 (10.8)	7 (4.1)
Separated	11 (2.7)	5 (2.9)
Educational status		
Cannot read and write	32 (13.9)	8 (4.7)
Only read and write	23 (10)	20 (11.8)
Grade 1–8	66 (28.6)	47 (27.6)
Grade 9–12	46 (19.9)	45 (26.5)
Diploma	51 (22.1)	28 (16.5)
First degree and above	13 (5.6)	22 (12.9)
Occupational status		
Government employee	64 (29.8)	45 (31)
Merchant	38 (17.7)	31 (21.4)
Private employee	58 (27)	59 (40.7)
Jobless	36 (16.7)	9 (6.2)
House wife	19 (8.8)	1 (0.7)
Monthly income, birr		
<600	45 (19.5)	18 (10.6)
600–2000	98 (42.4)	74 (43.5)
2001–4000	47 (20.3)	29 (17.1)
4001–6000	20 (8.7)	24 (14.1)
>6000	21 (9.1)	25 (14.7)
Mean	2715.68	

**Table 2:** Anthropometric values and behavioral characteristics of study subjects, Akaki Kality, Addis Ababa, Ethiopia, 2018.

Variable		Women, *n* (%)	Men, *n* (%)
Weight, kg			
≤54		39 (17.2)	16 (9.4)
55–64		60 (26.4)	39 (22.9)
≥65		128 (56.4)	115 (67.6)
Mean		67.03	
Median		67	
Height in meters			
≤1.54		41 (17.7)	9 (5.3)
1.55–1.64		72 (31.2)	21 (12.4)
≥1.65		118 (51.1)	140 (82.4)
Mean		1.65	
Median		1.68	
Body mass index, kg/m^2^			
<18.5		10 (4.3)	9 (5.3)
18.5–25		128 (55.4)	102 (60)
≥25		93 (40.3)	59 (34.7)
Mean		24.27	
Median		24.30	
Cigarette smoking			
Yes		76 (32.9)	75 (44.1)
No		155 (67.1)	95 (55.9)
Amount of smoking			
<1 pack per day		7 (35)	11 (39.3)
Only 1 pack per day		11 (55)	14 (50)
2 packs per day		2 (10)	2 (7.1)
≥3 packs per day		0	1 (3.6)
Alcohol drinking			
Yes		20 (8.7)	29 (17.1)
No		211 (91.3)	141 (82.9)
Amount of alcohol drinking			
<1 drink per day		2 (2.8)	9 (11.1)
Only 1 drink per day		19 (26.4)	26 (32.1)
>1 drink per day		51 (70.8)	46 (56.8)
Khat chewing			
Yes		20 (8.7)	24 (14.1)
No		211 (91.3)	146 (85.9)
Frequency of khat chewing			
Sometimes per week		11 (57.9)	15 (55.6)
Daily		6 (31.6)	9 (33.3)
2 times daily		0	1 (3.7)
≥3 times daily		2 (10.5)	2 (7.4)
Practicing regular exercise			
Yes		103 (44.6)	87 (51.2)
No		128 (55.4)	83 (48.8)
Walking as a regular exercise			
Yes		83 (35.9)	63 (37.1)
No		148 (64.1)	107 (62.9)
Running as a regular exercise			
Yes		14 (6.1)	13 (7.6)
No		217 (93.9)	157 (92.4)
Jogging as a regular exercise			
Yes		7 (3)	11 (6.5)
No		224 (97)	159 (93.5)
Swimming as a regular exercise		
Yes		5 (2.2)	2 (1.2)
No		226 (97.8)	168 (98.8)

**Table 3:** Status of hypertension in the study populations, Akaki Kality, Addis Ababa, Ethiopia, 2018.

Variable	Women, *n* (%)	Men, (*n* %)
Systolic blood pressure, mmHg		
<90	2 (0.9)	0(0)
90–139	205 (88.7)	148 (87.1)
≥140	24 (10.4)	22 (12.9)
Mean	120.88	
Median	120	
Diastolic blood pressure, mmHg		
<60	0	0
60–89	209 (90.5)	148 (87.1)
≥90	22 (9.5)	22 (12.9)
Mean	79.87	
Median	80	
Prevalence of hypertension		
Total	26 (11.3)	30 (17.6)
Type of hypertension		
Isolated systolic	6 (2.6)	4 (2.4)
Isolated diastolic	4 (1.7)	4 (2.4)
Stage of hypertension		
Stage I	15 (6.5)	18 (10.6)
Stage II	9 (3.9)	10 (17)
Stage III (severe)	2 (0.9)	2 (1.2)

**Table 4:** Factors affecting hypertension (bivariate and multivariate analyses), Akaki Kality, Addis Ababa, Ethiopia, 2018.

Variable	*β*	SE	*P*		OR		95% CI for AOR	
			Crude	Adjusted	COR	AOR	Lower	Upper
Alcohol drinking-yes	2.472	0.608	0.001	0.001	32.30	11.844	3.596	39.014
No (reference)								
Cigarette smoking-yes	2.804	0.633	0.001	0.001	93.33	16.511	4.775	57.084
No (reference)								
Khat chewing-yes	1.932	0.694	0.001	0.005	43.57	6.905	1.773	26.889
No (reference)								
BMI (kg/m^2^) >25	0.277	0.071	0.001	0.001	1.404	1.320	1.149	1.516
<18.5 (reference)								
Age >44 years	0.039	0.018	0.001	0.028	1.051	1.040	1.004	1.077
<35 years (reference)								

## Data Availability

The data that support the findings of this study are available on request from the corresponding author.
